# Immediate effects of cervicothoracic junction mobilization versus thoracic manipulation on the range of motion and pain in mechanical neck pain with cervicothoracic junction dysfunction: a pilot randomized controlled trial

**DOI:** 10.1186/s12998-020-00327-4

**Published:** 2020-08-07

**Authors:** Shriya Joshi, Ganesh Balthillaya, Y. V. Raghava Neelapala

**Affiliations:** Department of Physiotherapy, Manipal College of Health Professions, Manipal Academy of Higher Education, Manipal, Karnataka India

**Keywords:** Cervical pain, Manual therapy, Manipulative therapy

## Abstract

**Background:**

Cervicothoracic (CT) junction hypomobility has been proposed as a contributing factor for neck pain. However, there are limited studies that compared the effect of CT junction mobilization against an effective intervention in neck pain. Thoracic spine manipulation is a nonspecific intervention for neck pain where remote spinal segments are treated based on the concept of regional interdependence. The effectiveness of segment-specific spinal mobilization in the cervical spine has been researched in the last few years, and no definite conclusions could be made from the previous studies. The above reasons warrant the investigation of the effects of a specific CT junction mobilization against a nonspecific thoracic manipulation intervention in neck pain. The present study aims to compare the immediate effects of C7-T1 Maitland mobilization with thoracic manipulation in individuals with mechanical neck pain presenting with CT junction dysfunction specifically.

**Methods:**

A randomized clinical trial is conducted where participants with complaints of mechanical neck pain and CT junction dysfunction randomly assigned to either C7-T1 level Maitland mobilization group or mid-thoracic (T3-T6) manipulation group (active control group). In both the groups, the post graduate student (SJ) pursuing Master’s in orthopedic physiotherapy delivered the intervention. The outcomes of cervical flexion, extension, side flexion & rotation range of motion (ROM) were measured before & after the intervention with a cervical range of motion (CROM) device. Self-reported pain intensity was measured with the numerical pain rating scale (NPRS). The post-intervention between-group comparison was performed using a one-way ANCOVA test.

**Results:**

Forty-two participants with mean age CT junction group: 35.14 ± 10.13 and Thoracic manipulation group: 38.47 ± 11.47 were recruited for the study. No significant differences in the post-intervention baseline adjusted outcomes of cervical ROM & self-reported pain intensity were identified between the groups after the treatment (p = 0.08, 0.95, 0.01, 0.39, 0.29, 0.27for flexion, extension, bilateral lateral flexion & rotations respectively) & neck pain intensity (p = 0.68). However, within-group, pre, and post comparison showed significant improvements in cervical ROM and pain in both groups.

**Conclusion:**

This preliminary study identified that CT junction mobilization is not superior to thoracic manipulation on the outcomes of cervical ROM and neck pain when level-specific CT junction mobilization was compared with remote mid-thoracic manipulation in individuals with mechanical neck pain and CT junction dysfunction.

**Trial registration:**

CTRI: 2018/04/013088, Registered 6 April 2018, http://ctri.nic.in/Clinicaltrials/pmaindet2.php?trialid=24418

## Background

Neck pain ranks 4th among the global causes of disability-adjusted life years, with a prevalence ranging from 30 to 50% in the general population [1, 2]. The causative mechanisms for neck pain are dependent upon various contributing factors and rarely implicate a single anatomical structure [2]. Hence, the majority of neck pain complaints are classified as nonspecific/mechanical neck pain, which is defined as the neck pain that increases with cervical spine movements in this study.

Restricted cervical spine mobility is an impairment frequently associated with neck pain [3]. Previous kinematic data has shown that the upper thoracic spine contributes significantly to the overall cervical movements [4]. The cervicothoracic (CT) junction is the transitional segment between the mobile lordotic cervical and less mobile kyphotic thoracic spines and hence a potential region for stiffness [5]. Reduction in cervicothoracic junction mobility has been proposed to cause neck pain, headaches & upper limb pain [6]. An altered mobility pattern termed “Inverse C7-T1 relationship” was shown to be associated with neck pain, thus suggesting the need to normalize CT junction mobility in individuals with neck pain [7]. Improving the mobility of the CT junction reduces the demand for movement in the mid and lower cervical segments, thereby reducing the stress on the cervical spine. In addition, CT junction has been considered a safe segment to perform mobilizations in neck pain [8].

Spinal mobilizations of the specific dysfunction segment or the remote segment are commonly used for the treatment of neck pain [9]. Despite the proposed role of CT junction hypomobility in causing neck pain, very few studies have investigated the effectiveness of CT junction mobilization in neck pain. A quasi-experimental trial by Creighton et al., [8] compared 2 mobilizations (gliding and distraction) techniques applied to the C7-T1 segment and reported an increased rotation range of motion (ROM) and reduced pain intensity after a single treatment session of both techniques. Another pre-post single group trial delivered 3 sessions of Mulligan’s Sustained Natural Apophyseal Glides (SNAGs) explicitly to the cervicothoracic region (C5-T4 levels), where only one participant received SNAGs to the C7 level [10]. A recent study by Kim & Kim [11] compared cervicothoracic mobilizations (C7-T3) with upper cervical mobilizations and identified superior outcomes in the cervicothoracic mobilization group. Therefore, there are limited high quality randomized controlled trials that have specifically investigated the effectiveness of CT junction mobilization and compared with an effective active intervention in neck pain.

Thoracic spine manual therapy has been commonly used for the treatment of neck pain based on the concepts of regional interdependence and neurophysiological effects of manual therapy [12]. Recent systematic reviews have concluded that thoracic manipulation is superior to thoracic mobilization on pain and disability in the short term [13, 14]. Due to the limited number of studies on CT junction mobilization, a randomized clinical trial was conducted to explore the immediate effects of CT junction mobilization in comparison with thoracic spine manipulation in mechanical neck pain patients. Specifically, the trail has been conducted on patients with CT junction dysfunction to determine the need for level-specific mobilization in the cervical spine. Thoracic spine manipulation has been chosen as an active control treatment group, as previous reports have reported significantly superior outcomes with thoracic manipulation in mechanical neck pain [13, 14].

## Methods

### Participants

The study was a randomized clinical trial (1:1 ratio) carried out in Kasturba Hospital (KH), Manipal, on patients referred to the physiotherapy department for the management of mechanical neck pain. Ethical approval was obtained from the Institutional Ethics Committee, and the trial was registered in the Clinical Trials Registry of India (CTRI; Registration number: 2018/04/013088). All the participants signed a written informed consent to participate in the study.

### Inclusion criteria

Individuals with a primary complaint of acute or chronic neck pain between the age of 18 to 60 years were included. Neck pain was defined as ‘*pain perceived along the posterior aspect of the neck, from superior nuchal line to the first thoracic vertebra, with the absence of any neurologic signs & specific pathologies*.’ Neck pain individuals with moderate to severe pain intensity, i.e., ≥4/10 on a numeric pain rating scale (NPRS) with cervicothoracic junction dysfunction were included in the trial. For the assessment of CT junction dysfunction, passive accessory intervertebral movements (PAIVMs) were performed at each cervical and the T1 segments. The participants were positioned in prone, and the intervertebral movement was assessed by delivering posteroanterior pressure using the tips of both thumbs against the spinous process in an oscillatory manner. The amount of segmental mobility (normal, hypo, or hypermobile) and pain provocation were assessed. This method of identifying the symptomatic segment in the cervical spine was determined to have acceptable reliability [15]. Subjects with pain provocation and reduced mobility at the CT junction segment were included in the study.

### Exclusion criteria

Patients were not considered eligible for inclusion if they reported a history of recent significant trauma, previous spine surgery, presence of any red flags, or pregnancy. Patients were excluded if neck pain was associated with cervical radiculopathy, whiplash injuries, severe headaches, cervical spine fracture, or vertebrobasilar insufficiency.

### Randomization & blinding

The participants were randomized into either the cervicothoracic junction mobilization group or the thoracic manipulation group. The random allocation of the participants to the groups was carried out by another individual not involved in the study procedures and the allocation concealment was ensured by opening the sealed envelopes and revealing the group to which the participant has to be allocated. Block randomization was done with seven blocks of 6 participants (n = 42) involving a computer-generated random sequence. Individual paper slips with the random assignment (3 slips each for group A and group B) were prepared and placed in a separate opaque envelope for each block. The outcome assessor evaluating the baseline & post-intervention outcome measures was blind to the group allocation of the participants, and the intervening therapist was not aware of the baseline scores of the participants. However, blinding of the patients and the treatment provider to the intervention was not possible.

### Outcome measures

The outcome assessor measured the cervical range of motion and neck pain intensity, before & 30 min after the intervention.

#### Cervical range of motion

The active ROM in the cervical spine was assessed with the Cervical Range of Motion (CROM) device. The CROM device is an instrument used across studies to measure ROM of the cervical spine, with good intra-rater reliability (0.7–0.9) & inter-rater reliability (0.8–0.87) [16] and excellent validity [17]. A minimal detectable change of 10° in the cervical rotation ROM was considered significant in the study [18, 19].

The cervical ROM measurements were performed with the participants sitting on a chair with back supported until the mid-thoracic level, with instructions to sit in an upright posture. CROM device along-with the magnetic neckpiece was appropriately placed on the participant’s head. A neutral head & neck position was established; to minimize variations in ROM that can arise due to head and neck postures. Participants were then instructed to perform active neck movements up to the point of pain provocation or until the maximum extent of the mobility. Participants were asked to perform flexion with chin tucked in, and the extension movement was started with the chin raise. Instructions were given to keep shoulders stationary, to avoid the thoracic spine movement. Manual stabilization of the contralateral shoulder was done by the outcome assessor during lateral flexion and rotation measurements. The ROM was recorded in degrees from the relevant inclinometer once the participant completed the range of motion. The dial of the inclinometer was manually adjusted to ‘zero’ before the participant performed the rotation movement [14, 15].

#### Pain intensity

The Numerical Rating Scale (NRS) was used to assess the self-reported pain intensity during the most painful neck movement. The participants were asked to rate the NRS score verbally after explaining to them that a score of zero equals ‘no pain,’ and the maximum score of 10 implies ‘unbearable pain.’ NRS was found to have fair to moderate test-retest reliability and satisfactory responsiveness in the mechanical neck pain population [20].

### Interventions

In both the groups, the post graduate student (SJ) pursuing Master’s in orthopedic physiotherapy delivered the intervention supervised by the faculty (GB, YVR) with a combined experience of 20 years in orthopedic physiotherapy.

#### CT junction mobilization

The participants received direction-specific Maitland mobilization to the C7-T1 level, according to their primary movement restriction (for flexion-extension restriction- central PA glide, for rotation restrictions-unilateral PA glide). The therapist decided the grade of mobilization according to severity, intensity & nature of the patient’s pain. The mobilizations were delivered in the prone position, with the patient’s forehead supported on his palms. The duration of mobilization was 30-s bouts given for three sets. For central PA glide, a central pressure angled towards the participant’s head was given with overlapping thumbs of the therapist placed on the spinous process of C7. The therapist’s thumbs were placed on the posterior surface of the articular process to be mobilized (C7 on the side of restriction), and anteriorly directed oscillatory pressure was applied for unilateral PA mobilization [21].

#### Thoracic manipulation

Participants in this group received a high-velocity low amplitude (HVLA) thrust at the mid-thoracic spine (T3-T6). The level of manipulation was decided by hypo mobility assessed with PAIVM testing [22]. Thrust manipulation was delivered in the prone position, with the therapist’s hands over the zygapophyseal joints of the hypo mobile vertebra. A single HVLA was performed, and if the audible cavitation was not achieved, a second thrust was given at the same level. After the intervention in both the groups, the treatment provider interviewed the participants to identify any potential adverse effects of the intervention.

### Statistical analysis

The sample size calculation was performed considering cervical ROM as the primary outcome measure and identified as 21 participants in each group. Based on the earlier reports [18, 19] on minimal detectable change (MDC) in cervical rotation range, a 10° between group post-intervention difference for the cervical rotation ROM and standard deviation of 10° (exceeding the value reported previously [18] was considered for the two-tailed sample size calculation at 80% power and level of significance as 0.05. As the study included acute neck pain participants, post treatment symptom exacerbation and a probable 30% dropout for post intervention outcome measurement was anticipated and the final sample size (n = 42) included the dropout rate. The data analysis was performed using SPSS version 16. The Kolmogorov-Smirnov test was used to assess the normality of the data. The comparison of post-intervention outcomes between the two groups was performed with a one-way ANCOVA test with baseline outcomes as the covariates, and the adjusted mean values were reported. The within-group changes in the outcome measures were compared using a paired t-test.

## Results

**Fig. 1:**
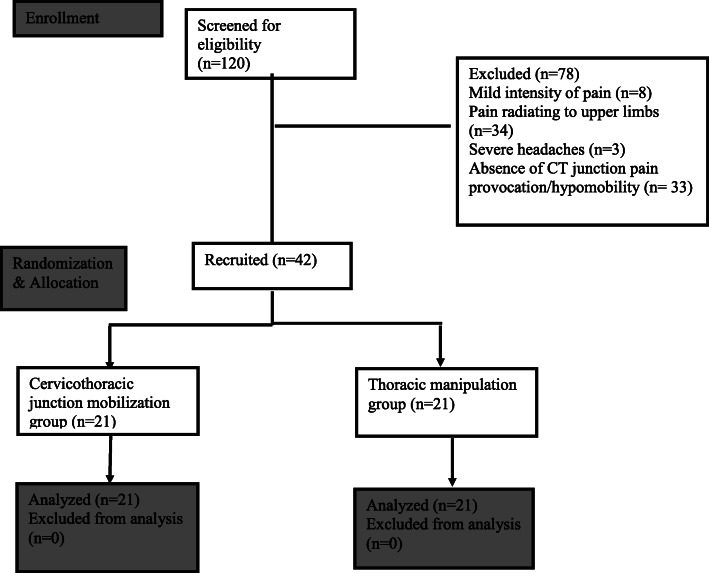
Participant Flow Diagram

A total of 120 participants were screened for eligibility & 42 participants were recruited in the study. Overall, 59 participants were identified to have CT junction dysfunction among the 120 individuals screened, suggesting a frequency of nearly 50%. Figure 1 summarizes the participant recruitment. Table 1 describes the demographic characteristics and baseline outcome measures of the participants.

**Table 1:** Participant demographics (n = 42)

	CT junction mobilization group (n = 21)	Thoracic manipulation group (n = 21)
Age (in years) *	35.14 ± 10.13	38.47 ± 11.47
Gender +	13 (62%) male, 8 (38%) female	10 (48%) male, 11 (52%) female
BMI (in kg/m2) *	23.57 ± 3.24	26.09 ± 2.75
Duration of pain +	Acute- 4 (19%) Subacute −5 (24%) Chronic −12 (57%)	Acute- 2 (9%) Subacute–6 (29%) Chronic-13 (62%)

*mean and standard deviation

+frequencies

### Inter-group comparison

The one-way ANCOVA analysis for the post-intervention baseline adjusted mean outcome comparison showed no statistically significant differences between the groups (p value > 0.05). The post intervention scores, and the base line adjusted mean differences (95% confidence interval) are shown in Table 2. The mean differences are below the accepted MDC value for the cervical ROM.

**Table 2:** Comparison between the groups (unadjusted means)

Outcome	CT junction mobilization group (mean ± SD)			Thoracic manipulation group (mean ± SD)			Adjusted mean difference (95% CI)	
	Baseline	Post treatment	p value	Baseline	Post treatment	p value		p value
Flexion	49.95 ± 8.59	54.52 ± 9.13	< 0.01	52.80 ± 11.88	55.28 ± 10.46	0.01	2.36 (− 0.9–5.6)	0.15
Extension	60.85 ± 13.15	64.42 ± 13.46	0.02	53.66 ± 10.51	58.09 ± 8.20	< 0.01	0.28 (− 3.2–3.8)	0.87
L SF	38.52 ± 9.20	41.33 ± 9.99	0.05	37.85 ± 9.18	41.19 ± 8.78	< 0.01	- 2.21 (− 4.9–0.5)	0.11
R SF	40.14 ± 10.38	41.66 ± 10.79	0.01	42.09 ± 11.42	42.09 ± 11.42	< 0.01	- 1.06 (− 4.2–2.1)	0.50
L ROT	61.80 ± 10.91	64.42 ± 12.36	< 0.01	57.90 ± 12.18	61.95 ± 14.10	0.02	1.42 (− 2.5–5.3)	0.47
R ROT	56.57 ± 10.06	59.90 ± 10.13	0.01	51.33 ± 15.85	57.71 ± 14.63	< 0.01	0.28 (− 3.4–3.9)	0.87
NRS	5.52 ± 1.47	4.33 ± 1.95	< 0.01	6.52 ± 1.80	5.23 ± 1.54	< 0.01	− 0.12 (− 0.9–0.6)	0.75

(L-left, R-right, SF-side flexion, ROT-rotation, SD- standard deviation; NRS- Numerical Rating Scale; Mean differences- CT junction mobilization group – Thoracic manipulation group)

### CT junction mobilization group

A statistically significant change was noticed in cervical flexion, extension, left side flexion & bilateral rotation ROM post-treatment with cervicothoracic mobilization (p-value < 0.05). However, the mean differences did not exceed the MDC for cervical ROM. Similarly, a statistically significant reduction in pain score was also obtained post-treatment (p-value< 0.01; and mean difference = 1.19).

### Thoracic manipulation group

In this group, statistically significant change was identified in the cervical ROM (p value < 0.05) and also in the pain scores (p-value = < 0.01; mean difference = 1.28). Similar to the CT junction group the pre post improvements did not exceed the MDC values for cervical ROM.

## Discussion

This preliminary RCT targeted the treatment of CT junction dysfunction in mechanical neck pain using either specific (CT junction) or nonspecific (mid-thoracic) joint mobilization/manipulation, respectively. Improvements in outcomes of pain intensity and cervical flexion, extension, and rotation ROM were identified after a single session of both C7-T1 level direction-specific mobilization and mid-thoracic manipulation. However, the comparison of the two manual therapy techniques revealed no significant differences between the two groups: for both cervical ROM and pain intensity. In addition, no adverse effects were reported in any of the treatment groups.

Few researchers have proposed that CT junction, being a transitional segment, is subjected to significant stress, and compressive loads and limited CT junction mobility contributes to neck pain [5]. CT junction mobilizations delivered in this study could have reduced the stiffness at this segment and increased the overall cervical ROM. The increase in cervical ROM (in degrees) is almost equivalent to the previous studies after a single session of CT junction mobilization [8, 11]. The thoracic manipulation group received treatment to an adjacent interdependent spinal region and demonstrated improvements in the study. According to the model of regional interdependence, dysfunction in one area can be influenced by the dysfunction of adjacent body segments [22]. The thoracic and cervical spines are biomechanically related, as researchers have found an association between thoracic kyphosis, thoracic mobility, and neck pain [23]. The improvements in the thoracic manipulation group could be due to improved mobility in thoracic spine. On the other hand, a recent biomechanical study on small sample showed that forces applied during thoracic manipulation at the T7 level extend to the cervical region as well [24]. Thus, the thoracic manipulation might have influenced the CT junction dysfunction among the participants causing improvements in ROM in this group. However, the changes obtained in the cervical ROM in both the groups did not exceed the minimal detectable change reported in the literature [18.19]. The probable reason for small improvements could be the application of only a single session of intervention.

Along with the likely biomechanical changes, neurophysiological effects of mobilization and manipulation could also have contributed to the improvements obtained. Spinal manual therapy has been shown to cause an immediate reduction in pain sensitivity by decreasing temporal summation, increasing remote pressure pain thresholds, and by reducing activation of the supraspinal regions involved in central pain processing [25]. Thus, the immediate changes obtained in the current study after a single session of spinal mobilization/manipulation to reduce pain and improve mobility could be explained by a combination of possible biomechanical, neurophysiological, and psychological effects [26].

Traditionally, spinal mobilizations had been targeted towards a specific spinal level of dysfunction, correcting the hypomobility and malalignment [27]. However, the need for spinal level-specific mobilization has been a topic of research interest in manual therapy for several years, with studies reporting conflicting results [28–30]. Two previous systematic reviews arrived at different conclusions regarding the effectiveness of segment-specific mobilization in the cervical pain in comparison with nonspecific mobilization [31, 32]. The review by Slaven et al. [31] concluded that segment-specific mobilizations are effective, and another review by Hidalgo et al., [32] stated no difference between segment-specific and nonspecific mobilizations in the cervical spine. Thus, no firm conclusion exists, regarding the need for level-specific mobilization in the cervical spine. The results of this preliminary study suggest level-specific mobilization is not superior to remote thoracic manipulation in mechanical neck pain and CT junction dysfunction participants.

### Limitations

In this study, only a manual assessment of the CT junction hypomobility was performed as no other standard measures of CT junction mobility were described in the literature. The objective measures of the cervicothoracic & thoracic spinal mobility could have provided additional information regarding the mechanics of the treatment effects. As this is the first study to explore the effects of CT junction mobilization in neck pain, it was not feasible from ethical regulations to deliver only a single intervention non-evidence-based intervention for a larger number of sessions to the participants. Hence the single session effects of CT junction mobilization were examined exclusively, which offer an initial evaluation of the benefits, safety, and dosage of CT junction mobilization technique. Also, the inclusion of both acute and chronic neck pain participants in the same study and not measuring the long-term effects, disability and function are additional limitations of the study that must be acknowledged.

### Clinical implications

The previous systematic reviews have concluded that thoracic spinal manipulation is more effective when compared with thoracic and cervical mobilization for the treatment of neck pain [2, 14]. This study demonstrated no greater advantage of level-specific mobilization over the manipulation of an interdependent region. The results support the finding of the Neck Task Force, 2016; that the type of neck mobilization may not impact the outcomes of the patient [9]. The results of the study suggest that mobilization of a regionally interdependent segment may also be beneficial when the mobilization of the specific local hypomobile segment is not possible due to superficial tenderness in neck cases with severe pain. The results of the study might be of interest in settings with high neck pain patient load and limited therapists, where single session physiotherapy consultations are practiced, though the long-term effects are yet to be explored. Though statistically significant within group differences were observed in both the groups, the magnitude of improvements is small, and a greater number of treatment sessions will be required to achieve a clinically significant outcome.

## Conclusion

This preliminary study is the first trial that investigated the effects of CT junction mobilization, specifically in participants with CT junction dysfunction with neck pain and compared with thoracic manipulation (active control intervention). The study identified that a single session of CT junction mobilization is not superior to thoracic manipulation in improving neck pain and in neck pain participants with CT junction dysfunction. This suggests that segment-specific mobilization may not be superior to the treatment of remote thoracic spine segments in patients with mechanical neck pain. However, future studies with larger sample size evaluating the long terms effects of CT junction mobilization in neck pain are necessary to confirm the findings of this study further.

## Data Availability

The datasets used and/or analyzed during the current study are available from the corresponding author on reasonable request.
